# Enhancing Sun-Dried Kelp Detection: Introducing K-YOLO, a Lightweight Model with Improved Precision and Recall

**DOI:** 10.3390/s24061971

**Published:** 2024-03-20

**Authors:** Zhefei Xiao, Ye Zhu, Yang Hong, Tiantian Ma, Tao Jiang

**Affiliations:** Fishery Machinery and Instrument Research Institute, Chinese Academy of Fishery Sciences, Shanghai 200092, China; xiaozhefei@frimi.ac.cn (Z.X.); zhuye@fmiri.ac.cn (Y.Z.); hongyang@fmiri.ac.cn (Y.H.); matiantian@fmiri.ac.cn (T.M.)

**Keywords:** deep learning, kelp, drying, attention mechanism, YOLOv5, optical images, UAV

## Abstract

Kelp, often referred to as a “sea vegetable”, holds substantial economic significance. Currently, the drying process for kelp in China primarily relies on outdoor sun-drying methods. Detecting kelp in the field presents challenges arising from issues such as overlapping and obstruction. To address these challenges, this study introduces a lightweight model, K-YOLOv5, specifically designed for the precise detection of sun-dried kelp. YOLOv5-n serves as the base model, with several enhancements implemented in this study: the addition of a detection head incorporating an upsampling layer and a convolution module to improve the recognition of small objects; the integration of an enhanced I-CBAM attention mechanism, focusing on key features to enhance the detection accuracy; the replacement of the CBS module in the neck network with GSConv to reduce the computational burden and accelerate the inference speed; and the optimization of the IoU algorithm to improve the identification of overlapping kelp. Utilizing drone-captured images of sun-dried kelp, a dataset comprising 2190 images is curated. Validation on this self-constructed dataset indicates that the improved K-YOLOv5 model significantly enhances the detection accuracy, achieving 88% precision and 78.4% recall. These values represent 6.8% and 8.6% improvements over the original model, respectively, meeting the requirements for the real-time recognition of sun-dried kelp.

## 1. Introduction

Kelp, a large brown algae rich in minerals and polysaccharides, serves as both a crucial nutritious food and a high-quality raw material for medicine and agricultural fertilizer [1,2]. Freshly harvested kelp typically undergoes two treatments: salting and light drying [3]. Specifically, freshly dried kelp first undergoes a drying treatment, followed by processing steps such as slitting. The prevalent method in China involves drying kelp on open sand, where workers spread fresh kelp on unobstructed sand before dawn, retrieve it before sunset, and turn it over at least once in the interim. This method, though labor-intensive, lacks real-time monitoring during the drying process and fails to retrieve kelp promptly in changing weather conditions. With the evolution of smart agriculture and smart ocean concepts, the marine science and technology field is incorporating intelligent and information technology applications [4,5,6]. The real-time monitoring and condition assessment of drying kelp are crucial for enhancing the processing efficiency and ensuring the product quality. Accurate kelp identification supports subsequent applications, like kelp counting and unmanned harvesting.

As computer technology advances, image processing and target recognition technologies are being increasingly applied in complex environments. Traditional target detection methods rely on color, texture, and shape features. Their processing speed is fast and less dependent on hardware, but they need to manually select features and have low robustness when disturbed by noise, which makes it difficult to meet the detection needs in complex environments [7,8,9]. In contrast, deep learning algorithms, despite having higher hardware requirements, automatically learn features from raw data, providing accurate detection in complex environments [10,11]. For instance, Tang et al. [12] optimized YOLOv3 for the real-time detection of crab molting in pike farming systems, achieving a 91% prediction accuracy even in muddy water. Sun et al. [13] improved the Faster RCNN algorithm for identifying flowers and fruits of unripe small tomatoes, reaching a model accuracy of 99.5%. Xiang et al. [14] enhanced YOLOX for precise soybean pod counting, achieving a 0.049 improvement in predicted vs. real values with a minor increase in the inference time.

Accurate identification becomes more challenging when target objects overlap and densely pack, leading to occlusion. Liu et al. [15] proposed a benthic organism detection method based on Faster RCNN. This method incorporates convolutional kernel adaptive units and designs an inverse convolutional feature pyramid structure in the backbone network. These enhancements effectively address the challenge of detecting small and densely packed organisms amidst overlap and occlusion, achieving an impressive accuracy rate of 96.7%. Wu et al. [16] improved the YOLOv4 model for apple detection in orchards. They replaced the original YOLOv4 backbone network with EfficientNet and added a convolutional layer to the three outputs, further refining the feature adjustment and extraction. The result was an impressive accuracy and recall of 97.43% and 95.52%, respectively.

While existing research focuses on underwater kelp recognition using remote sensing techniques [17,18,19], the recognition of kelp in the drying or processing stages is relatively limited. Given the numerous drying kelp instances, we propose using drones to capture images of sun-dried kelp and train a deep learning model for real-time and accurate recognition. This paper introduces a lightweight model, K-YOLO, based on YOLOv5-n, which is suitable for mounting on UAVs or other mobile devices, providing a technical basis for subsequent kelp counting, condition monitoring, unmanned management, and harvesting through aerial photography of drying fields by UAVs.

The main contributions of this paper include the following:

1. A new sun-dried kelp dataset: Capturing images of sun-dried kelp using UAVs, manual labeling, and data enhancement resulted in a dataset comprising 2190 images;
2. A new modified model: Addressing challenges such as overlapping, obscuring, kelp breakage, and interference factors, the modified model aims to improve the detection accuracy without sacrificing the inference speed;
3. A new attention mechanism and Intersection Over Union (IoU): Introducing a new attention mechanism (I-CBAM) and an improved αS-IoU algorithm enhances the model’s feature extraction ability, guiding it towards better convergence and effectively improving its accuracy.

## 2. Materials and Methods

### 2.1. Datasets

**Figure 1:**
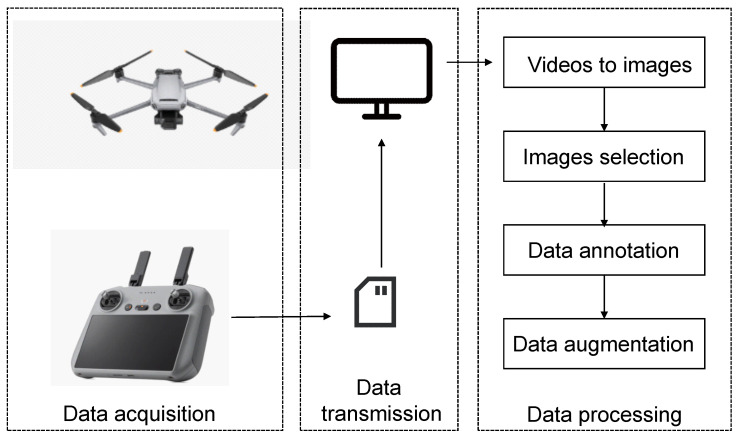
Dataset acquisition and processing in this research.

The experimental samples in this study consisted of kelp sourced from Xunshan Town, Rongcheng, Shandong Province, China, and data collection occurred from 25 May to 1 June 2023. A DJI Mavic 2 drone was employed to capture videos of sun-dried kelp on the sand, covering distances ranging from 3 to 40 m. These videos were then segmented into images of size 1920 × 1080, and a subset of 1095 images was selected to compose the dataset. The labeling of kelp locations in each image was manually performed using LabelImg. Given the high density and frequently overlapping nature of sun-dried kelp, instances where multiple kelps were challenging to distinguish were labeled collectively. Following the completion of the labeling, a txt file containing category and coordinate information was generated, and, simultaneously, an XML file was created for comparison with other algorithms. To enhance the model’s robustness and prevent overfitting during training, the dataset underwent augmentation through additive noise, random rotation, and random masking, resulting in an expansion to 2190 images. The dataset was then partitioned into training, validation, and test sets at a ratio of 7:2:1, comprising 1533, 438, and 219 images, respectively. Figure 1 shows the dataset acquisition and processing in this research.

### 2.2. Instrument Used for Data Acquisition

Considering factors such as camera investments, infrastructure costs, maintenance expenses, and the utility frequency of a fixed-positioned camera system, along with the outdoor working environment, the decision was made to utilize a drone for capturing images. This choice was motivated by the considerable size of the sun-dried kelp grounds, necessitating the camera to be positioned at a specific height above the ground for comprehensive image capturing. Additionally, the limitation posed by the UAV’s battery capacity can be mitigated through the use of exchangeable batteries and employing multiple drones. Equipped with an advanced omni-directional vision system and an infrared sensing system, the DJI Mavic 2 can hover and fly stably both indoors and outdoors, with auto-return and obstacle sensing, offering a range of user-friendly intelligent flight functions. The Maciv 2 can be flown by an operator with minimal training. The basic characteristics of the Mavic 2 are detailed in Table 1.

The Mavic 2 is equipped with a camera jointly developed with Hasselblad and a high-precision 3-axis gimbal to capture HD video or images. The remote control adopts OCUSYNCTM 2.0 HD transmission technology, which ensures a smooth 1080p HD transmission regardless of changes in flight attitude in a non-interference and unobstructed environment up to 10 km away. The camera parameters are shown in Table 2

### 2.3. Introduction to YOLOv5 Algorithm

The YOLO (You Only Look Once) series of algorithms [20,21,22] employ a single-stage detection model structure, processing the entire image simultaneously to achieve rapid and highly accurate target detection, recognition, or segmentation. YOLOv5 version 6.0 offers five distinct frameworks catering to varying requirements of accuracy and speed: YOLOv5n, YOLOv5s, YOLOv5m, YOLOv5l, and YOLOv5x, differing primarily in their width and depth.

**Figure 2:**
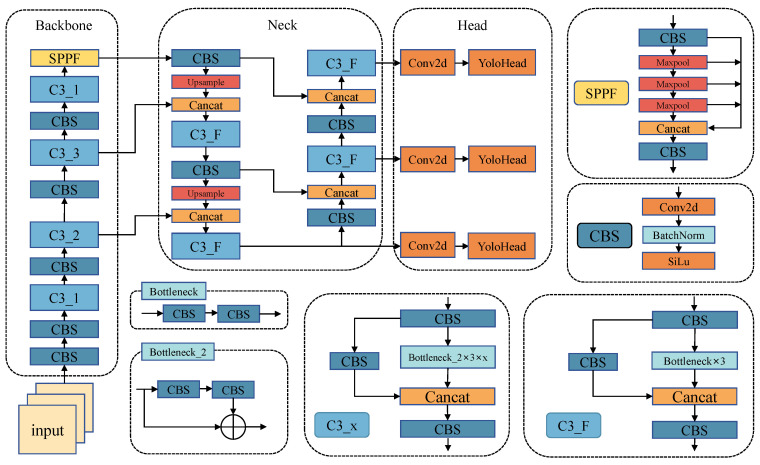
Structure of YOLOv5.

The YOLOv5 architecture comprises four primary components: the input, the backbone network, the neck network, and the detection head, as illustrated in Figure 2.

At the input stage, YOLOv5 employs the Mosaic algorithm for data augmentation, enhancing the training efficiency and model generalization by randomly selecting and stitching several samples into a single image after scaling, rotating, and cropping. Additionally, YOLOv5 employs an adaptive anchor strategy, continuously adjusting the preset anchor frame to better suit the current dataset with the aid of K-means and genetic learning algorithms. To further expedite inference, YOLOv5 utilizes adaptive image scaling to minimize redundant information when scaling the original image to a uniform size on input.

The backbone network, responsible for feature extraction, incorporates CBS, C3, and Spatial Pyramid Pooling-Fast (SPPF). The CBS module comprises convolutional, batch normalization, and activation layers in series. The C3 module enhances the model fusion capability by concatenating feature maps before and after the residual structure. The SPPF layer, based on Spatial Pyramid Pooling (SPP), improves upon it by connecting three maximum pooling layers in series and concatenating the outputs to extract maximal high-level semantic information and expand the receptive field.

The neck network consists of the Feature Pyramid Network (FPN) and the Path Aggregation Network (PAN). The FPN integrates feature maps at different scales through top–down connectivity, while the PAN employs bottom–up paths to further integrate feature maps of different scales, thereby enhancing the detection accuracy. The neck network effectively combines feature maps extracted by the backbone network at various scales.

The detection end, or “head” network, parses feature maps from the neck network and performs the classification, localization, bounding box regression, or segmentation of target objects. It comprises three branches for detecting objects of different sizes, with scales of 80 × 80, 40 × 40, and 20 × 20. The network employs the G-IoU [23] to compute loss between predicted and ground truth bounding boxes while applying non-maximum suppression (NMS) to eliminate redundant boxes.

### 2.4. K-YOLO

**Figure 3:**
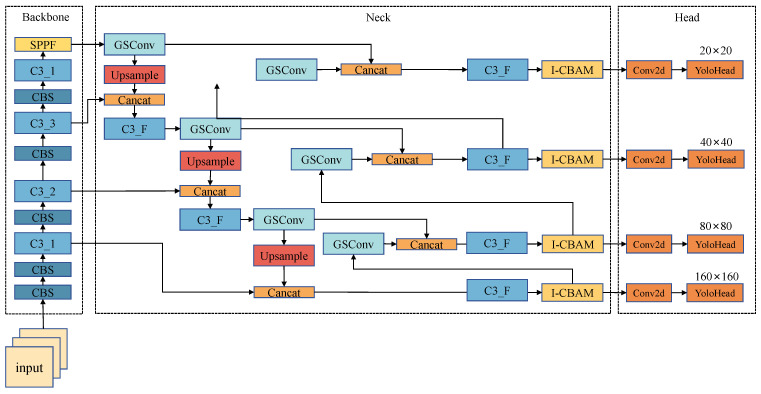
Structure of K-YOLO.

Considering the usage scenario considerations outlined in this study, the limitations of mobile hardware in supporting large networks led to the selection of YOLOv5-n, which boasts the smallest width and depth, as the benchmark model. Despite YOLOv5-n’s faster detection speed, it exhibits deficiencies in precision and accuracy. The outdoor sun-dried kelp environment, while relatively straightforward, presents challenges such as interference from water plants and debris, as well as issues like kelp stacking and mutual occlusion. These complexities escalate the difficulty of accurate recognition. In response, enhancements were introduced based on YOLOv5-n: firstly, the number of detection heads increased from 3 to 4, and a new upsampling layer and convolutional module were incorporated to broaden the global field of view and improve the detection of small objects. Subsequently, an advanced attention mechanism, I-CBAM, was integrated in front of the convolution module with 4 detection heads, aiming to focus the model’s attention on valuable feature layers and enhance the detection accuracy. Following that, the CBS module of the neck network was replaced with a lightweight GSConv module to reduce the computation and expedite the inference speed. Lastly, the G-IoU was substituted by the αS-IoU algorithm to enhance the localization ability for overlapping dense targets. These enhancements collectively form the K-YOLO model, tailored to meet the real-time detection demands of sun-dried kelp. The improved model’s structure is depicted in Figure 3. Further details on each improvement are provided in the subsequent sections.

### 2.5. Improvement of Small Target Detection Head

The size of the kelp in the image is directly linked to the flight altitude of the UAV. Given the variable altitude during UAV flight, the size of the target object undergoes significant variations. When the UAV operates at an altitude near 40 m, the resulting image portrays a dense arrangement of kelp, posing a considerable challenge to the model’s recognition capabilities. The original model’s P3 detection head faces limitations in small target detection due to the downsampling of the feature map by a factor of 8.

To address this issue and enhance the network’s coverage of target objects of varying sizes, as well as to improve the recognition accuracy for small targets, this paper introduces a small target detection head alongside the original three detection heads of YOLOv5. This addition involves inserting a CBS module after the second C3 module of the neck network. The feature maps, processed through an upsampling layer, are concatenated with the output from the first C3 module of the backbone network, creating a new detection head with a scale of 160 × 160.

This augmented detection head not only enhances the accuracy of small target recognition but also enables combination with feature maps from lower stages of the backbone network, preserving essential semantic information. Consequently, this design enlarges the global receptive field of the model, thereby improving the detection of overlapping or occluded objects.

### 2.6. Improvement of the Attention Mechanism

In the process of sun-dried kelp detection, the presence of surrounding water plants and debris significantly impacts the model’s accuracy. To mitigate environmental influence, this study introduces the attention mechanism, a proven method to enhance the model detection accuracy [24,25,26]. The attention mechanism achieves this by assigning different weights to features, prioritizing valuable features with larger weights and de-emphasizing less valuable ones with smaller weights, prompting the model to focus on critical features.

The CBAM (Convolutional Block Attention Module) [27] consists of two integral components: the channel attention and the spatial attention. It conducts maximum pooling and average pooling on the feature maps along spatial and channel dimensions, respectively. The generated weights, computed through convolution, are multiplied with the original feature map, producing a new feature map with attention markers [28]. The spatial attention guides the neural network to concentrate on essential pixel regions crucial for classification, while the channel attention prioritizes valuable feature map channels. The synergy of these components effectively enhances the model performance, addressing the limitations of convolutional networks that tend to focus solely on local information without capturing global context.

Two practical improvements are made to the CBAM for this application:

- The replacement of the activation function: Original activation function, ReLU, is replaced with Hardswish for a more stable training process and lightweight requirements. Explanation: ReLU can limit the expressive power of the model as some neurons are never activated. Swish was proposed, which has the properties of no having no upper bound with a lower term, being smooth and non-monotonic, and showing better results on deep models [29]. The formula of Swish is as follows:
  (1) $$Swish\left(x\right)=x*sigmoid\left(\beta x\right)=\frac{x}{1+e^{-\beta x}}$$
  where β is a variable parameter. However, Swish is computationally intensive and is not suitable for embedded and other miniature devices. Hardswish, a variation of Swish, offers smoother curves with less computational effort, making it suitable for lightweight operations [30].
  The formula of Hardswish is as follows:
  (2) $$Hardswish\left(x\right)=\left\{\begin{array}{cc} 0, & \;\mathrm{if}\;x\leq -3\\ x, & \;\mathrm{if}\;x\geq 3\\ x*(x+3)/6, & \;\mathrm{otherwise}\; \end{array}\right.$$
- Spatial channel enhancement: The original single convolutional layer in the spatial channel is replaced with a combination of convolutional and batch normalization layers. The subsequent sigmoid activation function is replaced by Hardswish. The addition of a batch normalization layer improves the spatial attention’s ability to capture information over long distances, enhancing the network generalization and stability during training. Hardswish, with a lower computational effort than Sigmoid, further accelerates the network operation.

**Figure 4:**
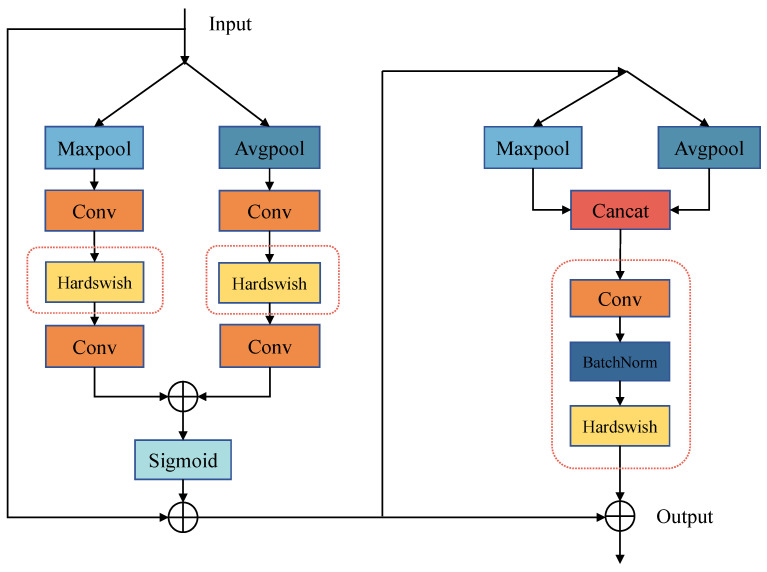
Struture of I-CBAM.

The structure of the improved I-CBAM attention mechanism is shown in Figure 4, with the enhanced components highlighted in red boxes. Notably, the I-CBAM module is placed at the neck network instead of the backbone network in this study, aiming to maximize retention of primary semantic information by the model.

### 2.7. Lightweight Module: GSConv

While the addition of a detection head expands the model’s receptive field and enhances the detection accuracy, it simultaneously increases the computational load and inference time due to the additional convolutional layer and the C3 module. To strike a balance between speed and accuracy, this study introduces the GSConv module to replace the convolutional layer of the CBS module in the neck network, aiming to reduce the model computation without compromising accuracy.

The GSConv module leverages a combination of convolutional layers and depthwise convolution to achieve high-precision detection with lightweight computation. In regular convolutional layers, each convolutional kernel is employed for all channels of the input, whereas depthwise convolution utilizes one convolutional kernel for each channel of the input. The outputs from depthwise convolution are concatenated, significantly reducing the computational workload [31].

The GSConv module processes the input through the convolutional layer, followed by depthwise convolution. Subsequently, the two results are spliced together, and a shuffle operation is performed to combine the channels corresponding to the outputs from both the regular and depthwise convolutions.

### 2.8. IoU Improvement in Loss Function

The loss function of YOLOv5 encompasses classification loss, bounding box regression loss, and confidence loss, with the IoU (Intersection over Union) utilized to assess the bounding box regression loss. This evaluation, quantifying the disparity between the predicted and ground truth bounding boxes, aims to guide the model towards optimal convergence. While YOLOv5 employs the G-IoU, which considers the ratio of the minimum closed area to the intersecting area between the predicted and ground truth bounding boxes, the SIoU (Spatial Intersection Over Union) introduces a refined loss function. This augmented loss function comprises four components: angular loss, distance cost, shape cost, and IoU loss, incorporating the vector angle between the ground truth box and the predicted bounding box.

**Figure 5:**
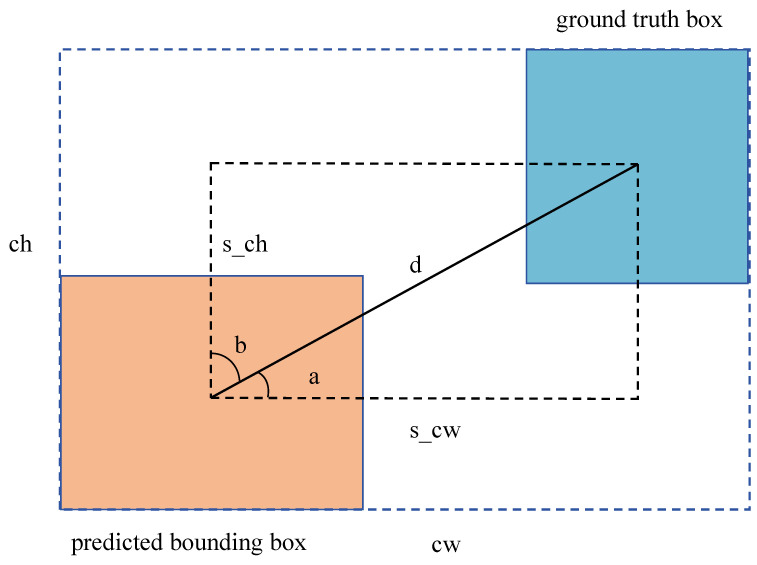
Diagram of S-IoU.

A schematic of the S-IoU is illustrated in Figure 5. If the angle a between the predicted bounding box and the ground truth exceeds 45°, the optimization prioritizes the angle b. The formula for angle loss is given by the following:

(3) $$AngleLoss=1-2*\sin^{2}\left(\arcsin\left(\frac{s\_ch}{d}\right)-\frac{\pi }{4}\right)$$

The height difference between the centers of the two rectangular boxes is denoted as s_ch, and d represents the distance between their centers.

The distance loss is computed by measuring the disparity between the widths and heights of the centroids of the two bounding boxes and those of the minimum bounding rectangle. The distance loss formula is given by the following:

(4) $$DistanceLoss=2-e^{-\gamma p_{x}}-e^{-\gamma p_{y}}$$

(5) $$p_{x}={\left(\frac{s\_cw}{cw}\right)}^{2}$$

(6) $$p_{y}={\left(\frac{s\_ch}{ch}\right)}^{2}$$

(7) γ=2−AngleLoss

The variable $p_{x}$ represents the square of the difference between the width of the center points of two boxes divided by the square of the width of the minimum bounding rectangle. Similarly, $p_{y}$ is calculated in the same manner, with the exception that it involves the height instead of the width. The angle loss is subtracted from 2.

The formula for shape loss is given by the following:

(8) $$ShapeLoss={\left(1-e^{-W_{w}}\right)}^{\theta }+{\left(1-e^{-W_{h}}\right)}^{\theta }$$

(9) $$W_{w}=\frac{\left|W-W^{gt}\right|}{max(W,W^{gt})}$$

(10) $$W_{w}=\frac{\left|H-H^{gt}\right|}{max(H,H^{gt})}$$

Here, *W*, *H*, $W^{gt}$ and $H^{gt}$ represent the width and height of the predicted bounding box and the ground truth, respectively. The parameter θ controls the degree of attention to the shape loss. To prevent an excessive focus on shape loss, potentially reducing the movement of the predicted bounding box, the authors specify a range of values for θ from 2 to 6. The chosen value in this study is 4.

The final formula for S-IoU is given by the following:

(11) $$S\_IoU=1-IoU+\frac{AngleLoss+DistanceLoss+ShapeLoss}{2}$$

He et al. introduce an approach known as the α-IoU, aiming to enhance bounding box regression by applying power transformations to existing IoU-based losses [32]. In an effort to improve the S-IoU’s robustness for noisy bounding boxes and achieve greater accuracy in overlapped bounding box regression, we drew inspiration from the α-IoU’s principles and improved the S-IoU by incorporating cubic values for its individual terms. We refer to this enhanced version as the αS-IoU, and its formula is presented in Equation (12):

(12) $$\alpha S\_IoU=1-IoU^{3}+{\left(\frac{AngleLoss+DistanceLoss+ShapeLoss}{2}\right)}^{3}$$

## 3. Experiments and Results

### 3.1. Experimental Environment and Parameter Setting

All experiments in this study were conducted on a system running Ubuntu 20.04, equipped with Intel Core i7 processors (Intel Corporation, Santa Clara, CA, USA) and a GeForce RTX 2080 Ti (NVIDIA, Santa Clara, CA, USA). The software frameworks employed for these experiments include Python 3.8, PyTorch 1.12, and CUDA 11.6.

During the training phase, hyperparameters were set as follows: The training was carried out for a total of 200 epochs. The initial 3 epochs comprised a warm-up training phase with a momentum factor of 0.8, while the subsequent epochs maintained a momentum factor of 0.937 with a learning rate fixed at 0.001. Prior to input into the model, all images were uniformly scaled to 640 × 640.

### 3.2. Evaluation Indicators

The evaluation indicators employed in this study to evaluate the models encompass the precision, recall, mean average precision at an IoU of 0.5 (mAP@0.5), mean average precision across IoUs of 0.5-0.95 (mAP@0.5-0.95), model size, number of floating-point operations (GFLOPs), and inference time per single image. The precision represents the ratio of correctly detected positive samples among all correctly identified samples, while the recall signifies the ratio of correctly detected positive samples among all actual positive samples. The respective formulas are presented below.

(13) $$P=\frac{TP}{TP+TN}$$

(14) $$Recall=\frac{TP}{TP+FN}$$

Here, *TP* denotes True Positive, the count of positive samples correctly classified; *FP* is False Positive, denoting samples originally negative but incorrectly identified as positive; and *FN* is False Negative, indicating samples originally positive but incorrectly classified as negative.

The mAP@0.5 denotes the average correctness rate across all classifications when the IoU is set to 0.5. The mAP@0.5-0.95 signifies IoUs ranging from 0.5 to 0.95 in increments of 0.05. The average of all the *mAPs* is computed using the following formula:

(15) $$AP=\int_{0}^{1}P\left(R\right)dR$$

(16) $$mAP=\frac{\sum_{i=1}^{C}AP_{i}}{C}$$

### 3.3. Algorithm Improvement Effect Comparison Test

**Figure 6:**
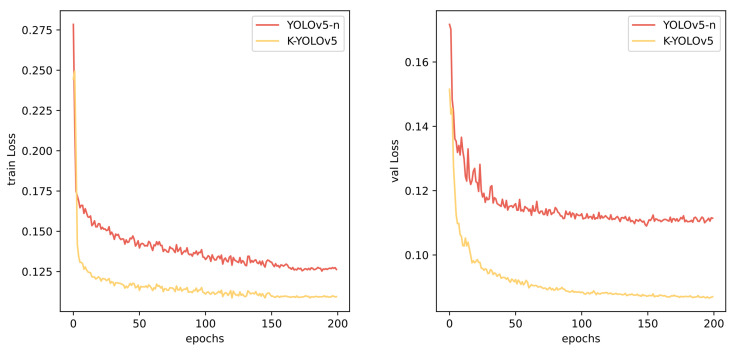
Training results.

Figure 6 illustrates the training and validation loss values during the training process for YOLOv5-n and K-YOLO. It is evident that K-YOLO converges more swiftly and attains lower loss values on both the training and validation sets. The validation loss values for YOLOv5-n exhibit more pronounced fluctuations. The final loss values for YOLOv5-n on the training and validation sets are 0.127 and 0.11, respectively, compared to 0.11 and 0.09 for K-YOLO.

**Figure 7:**
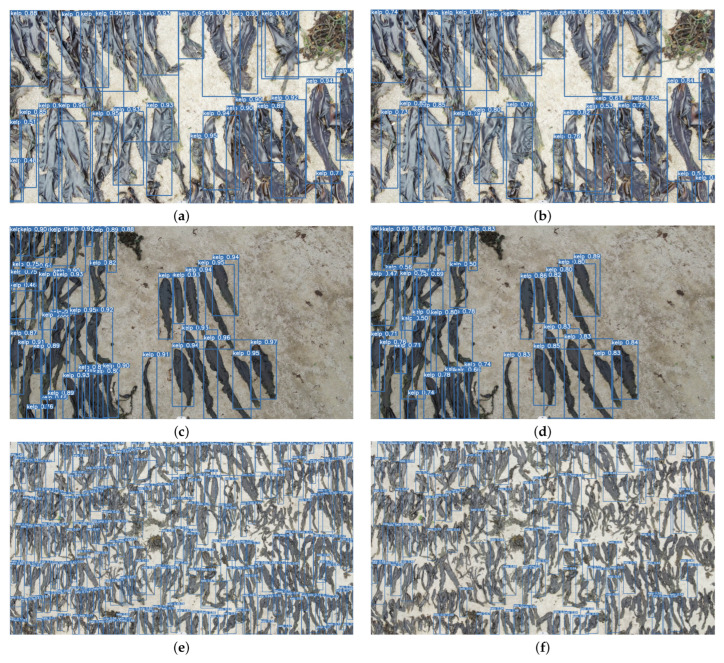
Training results: (**a**) detection result of K-YOLO at close range; (**b**) detection result of YOLOv5-n at close range; (**c**) detection result of K-YOLO at medium range; (**d**) detection result of YOLOv5-n at medium range; (**e**) detection result of K-YOLO at large range; (**f**) detection result of YOLOv5-n at large range.

Figure 7 displays the detection results of YOLOv5-n and K-YOLO at a close range (3–10 m), medium range (10–25 m), and long range (25–40 m). It is evident that the confidence scores of K-YOLO are generally higher than that of YOLOv5-n. YOLOv5-n exhibits several leakage detections in the long-range image, with some kelp not being recognized. In constrast, K-YOLO has fewer missed detections, validating the effectiveness of the improved method implemented in this study for kelp detection.

### 3.4. Ablation Experiments

To verify the effectiveness of the improvements proposed in this study and their impact on the model performance, ablation experiments were conducted using the same dataset. YOLOv5-n was used as the base model, with subsequent models incorporating specific improvements. Model1 extended YOLOv5-n by adding an additional detection head. Model2 augmented Model1 by integrating four I-CBAM attention modules into the neck network. Model3 substituted the CBS module in the neck network of Model2 with the GSConv module. Model4 replaced the G-IoU with the αS-IoU improvement proposed in this study, resulting in K-YOLO. All models were initialized with pre-trained weights from YOLOv5-n. Validation was performed on the test set, and the results are presented in Table 3.

The findings in Table 3 indicate that Model1 enhances the precision by 3.6% through the addition of a detection head compared to the baseline model, demonstrating the positive impact of increasing the detection scale and expanding the global receptive field on the model detection performance. However, this enhancement also resulted in a 21.9% increase in the GFLOPs and a 1.3 ms increase in the average single-image inference time. The integration of four I-CBAM attention modules into the neck network led to a 5.2% improvement in recall, accompanied by a slight increase in the GFLOPs and a 0.6 ms increase in the inference time. The introduction of the GSConv module reduced the GFLOPs, effectively minimizing the computational overhead and shortening the inference time by 1.2 ms while maintaining the model accuracy with a 1.3% improvement in the mAP@0.5 and mAP@0.5-0.95 each. Substituting the G-IoU with the αS-IoU resulted in enhancements across multiple metrics, including the model accuracy, recall, mAP@0.5, and mAP@0.5-0.95, with improvements of 1.6%, 2.4%, 1.3%, and 2.9%, respectively.

Thus, the lightweight improved model, K-YOLO, presented in this study significantly enhances model detection precision with improvements in the accuracy, recall, mAP@0.5, and mAP@0.5-0.95 by 6.8%, 8.6%, 4.9%, and 5.2%, respectively, compared to the original baseline model YOLOv5-n, while marginally increasing model complexity and inference time.

### 3.5. Comparsion of Different Attention Mechanisms

**Figure 8:**
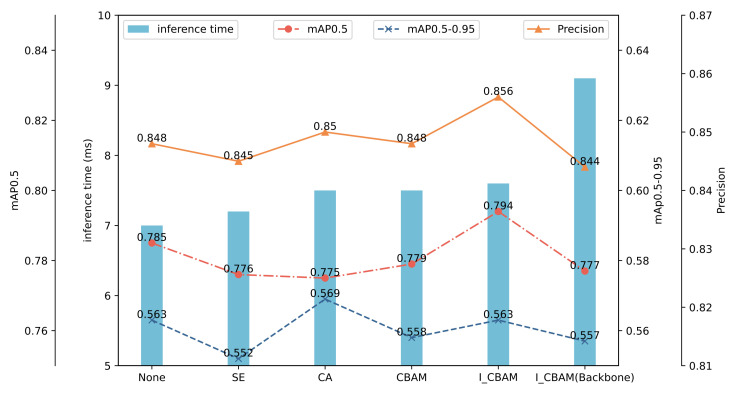
Comparison of different attention mechanisms.

To comprehensively investigate the impact of the I-CBAM and its insertion position on the model performance, this study explores the insertion of SE [33], CA [34], and CBAM attention mechanisms at the same position. Additionally, four I-CBAMs were inserted into the backbone network before each C3 module. The models underwent training using the same dataset and were subsequently tested, with the results depicted in Figure 8.

The SE module exhibited the least impact on the inference time, increasing it by only 0.3 ms. Conversely, both the CA and CBAM led to a 0.5 ms increase, while the I-CBAM in the neck network resulted in a 0.6 ms increment. The addition of the SE module resulted in decreases in the model precision, mAP@0.5, and mAP@0.5-0.95 by 0.3%, 0.9%, and 1.1%, respectively. The CA module yielded a 0.6% improvement in the mAP@0.5-0.95. In comparison to the CA module, the I-CBAM introduced in this study demonstrated a 0.6% and 1.9% greater improvement in the model precision and mAP@0.5, respectively. Furthermore, compared to the original CBAM module, the I-CBAM exhibited improvements of 0.8%, 1.5%, and 0.5% in the model precision, mAP@0.5, and mAP@0.5-0.95, respectively. Hence, the I-CBAM module in this study contributes the most significant overall improvement to the model.

Inserting the I-CBAM into the backbone network led to a reduction in the model evaluation indicators compared to its insertion into the neck network. The values of the precision, mAP@0.5, and mAP@0.5-0.95 were lower than the network without attention mechanisms by 0.4%, 0.8%, and 0.6%, respectively, and lower than the insertion into the neck network by 1.2%, 1.7%, and 0.6%. This may be attributed to the use of the attention mechanism in the primary feature extraction stage, compressing the space and channels of feature maps, leading to a loss of some semantic information and a reduction in the feature extraction capability. Additionally, inserting the I-CBAM into the backbone network increased the inference time to 11.1 ms, 0.6 ms more than inserting it into the neck network. This indicates that the introduction of the attention mechanism into the backbone network imposes a higher computational load.

### 3.6. Comparison of Different Lightweight Modules

To validate the efficacy of lightweight modules, this study conducts a comparative analysis between two modules: GhostConv [35] and GSConv. Both modules are incorporated into the same position, and the network is trained accordingly, with results presented in Table 4. The data reveal that both lightweight modules effectively reduce the model’s GFLOPs and overall size.

While the model utilizing GhostConv achieves a 0.2 ms reduction in the inference time compared to the GSConv-utilizing model, it experiences a 2.4% decrease in accuracy and a 4.5% reduction in recall. It is observed that both modules contribute to model lightweighting, yet GSConv demonstrates a higher accuracy improvement on the dataset utilized in this study, making it more suitable for sun-dried kelp detection.

### 3.7. Comparion of Different IoUs

This study delves deeper into assessing the impact of introduced enhancements, specifically validating the effectiveness of the C-IoU, S-IoU, and E-IoU on the test set. The results are presented in Table 5. In comparison to the original G-IoU (Model3 in Table 3), all these IoU algorithms demonstrate an enhancement in the model’s detection accuracy without exhibiting significant differences in the GFLOPs and inference time.

Among the IoU algorithms, the model outperforms others across all indicators, particularly excelling in the recall and mAP@0.5-0.95. Relative to the C-IoU, S-IoU, and E-IoU, the recall improves by 2.4%, 3.4%, and 1.5%, while the mAP@0.5-0.95 improves by 2.8%, 3.2%, and 2.2%, respectively. Consequently, the enhancements introduced in this paper prove most impactful in terms of improving the model performance, especially for detecting overlapping and dense target objects. Additionally, taking the cubic value of the single term in the S-IoU contributes to a better model convergence, thereby achieving a superior detection performance.

### 3.8. Comparison with Other Object Detection Models

**Figure 9:**
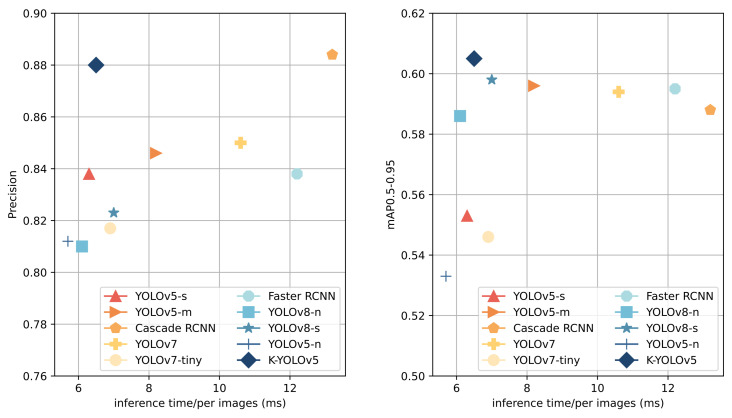
Comparison of different models.

In this study, a comprehensive comparison of widely used detection algorithms is conducted on the same dataset, encompassing one-stage detection models YOLOv5-s, YOLOv5-l, YOLOv7, YOLOv7-x, YOLOv7-tiny, YOLOv8-n, and YOLOv8-s, along with the two-stage detection models Faster RCNN and Cascade RCNN. Both Faster RCNN and Cascade RCNN employ ResNet50 as the backbone network, and all models are trained using pre-trained weights. To ensure fair testing, all models undergo validation on the test set after uniform resizing of input images to 640 × 640 pixels. The results are presented in Figure 9 and Table 6, highlighting the highest metric values and the shortest inference time in bold in Table 6.

Faster RCNN and Cascade RCNN, common two-stage detection models, exhibit distinct strengths. Cascade RCNN achieves the highest precision value among all algorithms at 0.884, while Faster RCNN leads in the recall and mAP@0.5 at 0.797 and 0.834, respectively. The proposed K-YOLO in this paper attains the highest mAP@0.5-0.95 value of 0.605, with its precision, recall, and mAP@0.5 closely approaching the best values. Its precision is only 0.4% lower than Cascade RCNN, and its recall and mAP@0.5 are slightly lower than Faster RCNN by 1.3% and 1.4%, respectively. Notably, the inference time of K-YOLO is significantly smaller than those of the two-stage models, being 6.7 ms and 5.7 ms faster than Cascade RCNN and Faster RCNN, respectively.

When compared with other one-stage YOLO series algorithms, K-YOLO emerges with superior metrics. Its precision, recall, mAP@0.5, and mAP@0.5-0.95 are 4.2%, 7.9%, 6.3%, and 5.2% higher than YOLOv5-s, respectively, with only a 0.2 ms increase in its inference time. Compared to YOLOv7, our method K-YOLO exhibits a 3% higher precision and 4.1 ms lower inference time. If compared with YOLOv8-s, K-YOLO shows improvements with a 5.7% higher precision, 1.5% higher recall, 0.7% higher mAP@0.5-0.95, and 0.5 ms lower inference time.

However, Table 4 reveals that, despite having the smallest GFLOPs of 4.8, K-YOLO is not as fast as YOLOv5-s and YOLOv8-n, which have GFLOPs of 15.8 and 8.9, respectively. The GFLOPs and inference time are not directly correlated metrics. We speculate that the lower memory utilization of the model, attributed to certain modules used in the improvement of K-YOLOv5, may be the reason. Ding et al. [36] demonstrated that depth-wise convolution increases the memory access cost. Additionally, the newly added concatenation operation in the detection branch does not increase the computational effort but leads to a longer memory access time. Therefore, the inference speed of K-YOLOv5 may not be as fast as ideal. Nonetheless, considering the balance between speed and accuracy, it can be asserted that K-YOLOv5 achieves a commendable equilibrium.

## 4. Conclusions and Future Work

In this study, we propose an improved sun-dried kelp detection method based on YOLOv5. Utilizing an unmanned aerial vehicle, we captured images of kelp in a sandy field, manually annotated them, and constructed a dataset comprising 2190 images. Using YOLOv5-n as the baseline model, we initially increased the number of detection heads from three to four to broaden the global receptive field and enhance the detection capability for small objects. Subsequently, the network’s feature-filtering capacity was bolstered by integrating the I-CBAM attention mechanism. The incorporation of the lightweight module GSConv reduced both the model’s computational load and inference time, thereby reducing the overall computational cost and facilitating potential embedded applications. Finally, the introduction of the algorithm further refined the model’s prediction accuracy for regression bounding boxes, improving its overall positioning precision.

Experimental results demonstrate the significant enhancement in model accuracy with the introduced I-CBAM attention mechanism. Compared to the SE, CBAM, and CA attention mechanisms, the model with the I-CBAM exhibited improvements of 1.1% and 1.8%, 0.6% and 1.9%, 0.8%, and 1.5% in its accuracy and mAP@0.5, respectively. Notably, the improvements presented in this paper excel in enhancing the accuracy of predicting regression bounding boxes, particularly in scenarios with dense overlapping target objects. In comparison to the C-IoU, S-IoU, and E-IoU, it elevates the mAP by 2.8%, 3.2%, and 2.2% for the mAP@0.5-0.95, respectively.

The enhanced K-YOLO model achieved a precision of 88% and a recall of 78.4%, marking improvements of 6.8% and 8.6% compared to the original model, respectively. When juxtaposed with other mainstream detection algorithms, including the one-stage algorithms YOLOv5, YOLOv7, and YOLOv8 and two-stage algorithms Cascade RCNN and Faster RCNN, K-YOLO strikes an effective balance between speed and accuracy.

This study offers valuable insights for enterprises and practitioners seeking to enhance the efficiency of kelp drying monitoring and management. The user-friendliness and maintenance advantage are evident as only drone flying skills need to be trained for the operator, and the model tuning frequency follows the kelp harvesting rhythm, which is once a year. Additionally, it lays a technical foundation for the future realization of fully mechanized and automated kelp drying processes.

The subsequent phase of this study will focus on the following areas:

1. To augment the dataset, various sources were utilized to acquire samples of kelp, encompassing cultivation across various regions and species. This involved partnerships with marine biology research institutes, leveraging their resources and expertise for sample acquisition. Concurrently, on-site inspections of kelp cultivation in disparate regions were coordinated with agricultural departments or farmers to ensure dataset diversity. Further diversification was achieved by gathering data under varying light and weather conditions, bolstering the model’s adaptability to unfamiliar external environments.
2. To enhance the model and prioritize the continual improvement of its detection accuracy and inference speed, a superior input image quality stands out as a crucial factor. Key contributors to image degradation encompass atmospheric dust, leading to noise in images; incompatible lighting conditions resulting in a low image resolution under weak lighting or lens flares in intense lighting situations; and the consistent movement of UAVs, impacting the image stability. Image preprocessing or enhancement techniques will be employed to alleviate the impact of atmospheric conditions, airborne dust, and other variables on the image quality. Additionally, sensor data will be integrated, such as GPS and inertial measurement units, and adaptive algorithms capable of dynamically adjusting to compensate for dynamic changes in UAV direction or distance from the target will be devised.
3. Application scenarios of the model, such as developing an application that can be deployed on drones or embedded devices for identifying, counting, and evaluating kelp in the drying process, will continue to be explored. This can enable the real-time monitoring and analysis of the kelp drying process, aiding kelp producers in better understanding the drying progress, identifying problems, and taking timely measures to ensure the drying effectiveness and kelp quality. A user-friendly mobile application or web-based interface will be developed that enables operators to easily use the model for identifying, counting, and evaluating dried kelp. The interface design should be simple and intuitive, providing clear operational guidelines and feedback to reduce the cost and time required for operator training.

## Figures and Tables

**Table 1:** Basic characteristics of UAV DJI Mavic 2.

Technical Parameters	Data
Takeoff weight	907 g
Maximum rising speed	5 m/s
Maximum horizontal flight speed	72 km/h
Maximum takeoff altitude	6000 m
Maximum wind resistance	level 5
Hovering accuracy	Horizontal: ±0.3 m; vertical: ±0.1 m

**Table 2:** Basic parameters of the camera.

Technical Parameters	Data
Image sensor	1^″^ CMOS
Number of effective pixels	20 Mpix
Maximal resolution	5472 × 3648
Angle of view	77°
Focal length	28 mm
Focus	1 m to infinity

**Table 3:** Ablation experiment.

Model	4-Head	I-CBAM	GSConv	αS-IoU	Precision	Recall	mAP@0.5	mAP@0.5-0.95	GFLOPs	Inference Time (ms)
YOLOv5-n	×	×	×	×	0.812	0.698	0.771	0.553	4.1	5.7
Model1	✓	×	×	×	0.848	0.702	0.785	0.563	5	7.0
Model2	✓	✓	×	×	0.856	0.754	0.794	0.563	5.1	7.6
Model3	✓	✓	✓	×	0.864	0.760	0.807	0.576	4.8	6.4
Model4	✓	✓	✓	✓	0.880	0.784	0.820	0.605	4.8	6.5

**Table 4:** Comparison between GSConv and GhostConv.

Model	Precision	Recall	mAP@0.5	mAP@0.5-0.95	File Size (MB)	GFLOPs	Inference Time (ms)
Model2 + GSConv	0.864	0.760	0.807	0.576	4.5	4.8	6.4
Model2 + GhostConv	0.840	0.715	0.776	0.564	4.5	4.8	6.2

**Table 5:** Comparison of different IoUs.

Model	Precision	Recall	mAP@0.5	mAP@0.5-0.95	GFLOPs	Inference Time (ms)
Model3 + -IoU	0.866	0.760	0.814	0.577	4.8	6.4
Model3 + S-IoU	0.872	0.750	0.802	0.573	4.8	6.5
Model3 + E-IoU	0.871	0.769	0.803	0.583	4.8	6.5
Model3 + αS-IoU	0.880	0.784	0.820	0.605	4.8	6.5

**Table 6:** Results of different models.

Model	Precision	Recall	mAP@0.5	mAP@0.5-0.95	GFLOPs	Inference Time (ms)
YOLOv5-s	0.838	0.705	0.757	0.553	15.8	6.3
YOLOv5-m	0.846	0.709	0.801	0.596	47.9	8.2
YOLOv7	0.85	0.78	0.82	0.594	103.2	10.6
YOLOv7-tiny	0.817	0.767	0.818	0.546	13	6.9
YOLOv8-n	0.81	0.768	0.817	0.586	8.9	6.1
YOLOv8-s	0.823	0.769	0.816	0.598	28.9	7.0
Cascade RCNN	**0.884**	0.71	0.792	0.588	60.3	13.2
Faster RCNN	0.838	**0.797**	**0.834**	0.595	88.2	12.2
K-YOLO	0.880	0.784	0.82	**0.605**	4.8	6.5

## Data Availability

All data supporting the results of this study are included in the manuscript and are available upon request.

## References

[1] Bennion M., Fisher J., Yesson C., Brodie J. (2019). Remote Sensing of Kelp (Laminariales, Ochrophyta): Monitoring Tools and Implications for Wild Harvesting. Rev. Fish. Sci. Aquac..

[2] Jiang T., Hong Y., Lu L., Zhu Y., Chen Z., Yang M. (2022). Design and experiment of a new mode of mechanized harvesting of raft cultured kelp. Aquac. Eng..

[3] Zhu Y., Hong Y., Jiang T., Yang M., Lu L., Yu Y. (2023). Design and Test of an Efficient Automatic Clip Seedling System for Raft Aquaculture Kelp. J. Mar. Sci. Eng..

[4] Qiu T., Zhao Z., Zhang T., Chen C., Chen C.L.P. (2020). Underwater Internet of Things in Smart Ocean: System Architecture and Open Issues. IEEE Trans. Ind. Inform..

[5] Li S., Li C., Yang Y., Zhang Q., Wang Y., Guo Z. (2020). Underwater scallop recognition algorithm using improved YOLOv5. Aquac. Eng..

[6] Liu P., Zhu B., Yang M. (2021). Has marine technology innovation promoted the high-quality development of the marine economy? ——Evidence from coastal regions in China. Ocean Coast. Manag..

[7] Li S., Zhang S., Xue J., Sun H. (2022). Lightweight target detection for the field flat jujube based on improved YOLOv5. Comput. Electron. Agric..

[8] Wang G., Ding H., Yang Z., Li B., Wang Y., Bao L. (2022). TRC-YOLO: A real-time detection method for lightweight targets based on mobile devices. IET Comput. Vis..

[9] Li X., Guo S., Gong L., Lan Y. (2023). An automatic plant leaf stoma detection method based on YOLOv5. IET Image Process..

[10] Hassan N., Ming K.W., Wah C.K. A Comparative Study on HSV-based and Deep Learning-based Object Detection Algorithms for Pedestrian Traffic Light Signal Recognition. Proceedings of the 2020 3rd International Conference on Intelligent Autonomous Systems (ICoIAS).

[11] Caroppo A., Leone A., Siciliano P. (2020). Comparison Between Deep Learning Models and Traditional Machine Learning Approaches for Facial Expression Recognition in Ageing Adults. J. Comput. Sci. Technol..

[12] Tang C., Zhang G., Hu H., Wei P., Duan Z., Qian Y. (2020). An improved YOLOv3 algorithm to detect molting in swimming crabs against a complex background. Aquac. Eng..

[13] Sun J., He X., Wu M., Wu X., Shen J., Lu B. (2020). Detection of tomato organs based on convolutional neural network under the overlap and occlusion backgrounds. Mach. Vis. Appl..

[14] Xiang S., Wang S., Xu M., Wang W., Liu W. (2023). YOLO POD: A fast and accurate multi-task model for dense Soybean Pod counting. Plant Methods.

[15] Liu Y., Wang S. (2021). A quantitative detection algorithm based on improved faster R-CNN for marine benthos. Ecol. Inform..

[16] Wu L., Ma J., Zhao Y., Liu H. (2021). Apple Detection in Complex Scene Using the Improved YOLOv4 Model. Agronomy.

[17] Uhl F., Bartsch I., Oppelt N. (2016). Submerged Kelp Detection with Hyperspectral Data. Remote Sens..

[18] Bewley M., Douillard B., Nourani-Vatani N., Friedman A., Pizarro O., Williams S. Automated species detection: An experimental approach to kelp detection from sea-floor AUV images. Proceedings of the Australasian Conference on Robotics and Automation (ACRA).

[19] Kim A.M., Olsen R.C., Lee K., Jablonski D. Using panchromatic imagery in place of multispectral imagery for kelp detection in water. Proceedings of the Ocean Sensing and Monitoring II.

[20] Redmon J., Divvala S., Girshick R., Farhadi A. (2016). You Only Look Once: Unified, Real-Time Object Detection. arXiv.

[21] Redmon J., Farhadi A. (2016). YOLO9000: Better, Faster, Stronger. arXiv.

[22] Redmon J., Farhadi A. (2018). YOLOv3: An Incremental Improvement. arXiv.

[23] Rezatofighi H., Tsoi N., Gwak J., Sadeghian A., Reid I., Savarese S. (2019). Generalized Intersection over Union: A Metric and a Loss for Bounding Box Regression. arXiv.

[24] Ma J., Lu A., Chen C., Ma X., Ma Q. (2023). YOLOv5-lotus an efficient object detection method for lotus seedpod in a natural environment. Comput. Electron. Agric..

[25] Bie M., Liu Y., Li G., Hong J., Li J. (2023). Real-time vehicle detection algorithm based on a lightweight You-Only-Look-Once (YOLOv5n-L) approach. Expert Syst. Appl..

[26] Li G., Huang X., Ai J., Yi Z., Xie W. (2021). Lemon-YOLO: An efficient object detection method for lemons in the natural environment. IET Image Process..

[27] Woo S., Park J., Lee J.Y., Kweon I.S. (2018). CBAM: Convolutional Block Attention Module. arXiv.

[28] Huang W., Huo Y., Yang S., Liu M., Li H., Zhang M. (2023). Detection of Laodelphax striatellus (small brown planthopper) based on improved YOLOv5. Comput. Electron. Agric..

[29] Ramachandran P., Zoph B., Le Q.V. (2017). Searching for Activation Functions. arXiv.

[30] Howard A., Sandler M., Chu G., Chen L.C., Chen B., Tan M., Wang W., Zhu Y., Pang R., Vasudevan V. (2019). Searching for MobileNetV3. arXiv.

[31] Chollet F. (2017). Xception: Deep Learning with Depthwise Separable Convolutions. arXiv.

[32] He J., Erfani S., Ma X., Bailey J., Chi Y., Hua X.S. (2022). Alpha-IoU: A Family of Power Intersection over Union Losses for Bounding Box Regression. arXiv.

[33] Hu J., Shen L., Albanie S., Sun G., Wu E. (2019). Squeeze-and-Excitation Networks. arXiv.

[34] Hou Q., Zhou D., Feng J. (2021). Coordinate Attention for Efficient Mobile Network Design. arXiv.

[35] Han K., Wang Y., Tian Q., Guo J., Xu C., Xu C. (2020). GhostNet: More Features from Cheap Operations. arXiv.

[36] Ding X., Zhang X., Ma N., Han J., Ding G., Sun J. (2021). RepVGG: Making VGG-style ConvNets Great Again. arXiv.

